# Cognition may link cortical IGFBP5 levels with motor function in older adults

**DOI:** 10.1371/journal.pone.0220968

**Published:** 2019-08-12

**Authors:** Aron S. Buchman, Lei Yu, Vladislav A. Petyuk, Chris Gaiteri, Shinya Tasaki, Katherine D. Blizinsky, Julie A. Schneider, Philip L. De Jager, David A. Bennett

**Affiliations:** 1 Rush Alzheimer’s Disease Center, Rush University Medical Center, Chicago, Illinois, United States of America; 2 Department of Neurological Sciences, Rush University Medical Center, Chicago, Illinois, United States of America; 3 Pacific Northwest National Laboratory, Richland, Washington, United States of America; 4 National Human Genome Research Institute, National Institutes of Health, Bethesda, Maryland, United States of America; 5 Department of Pathology (Neuropathology), Rush University Medical Center, Chicago, Illinois, United States of America; 6 Department of Neurology, Center for Translational & Computational Neuroimmunology, Columbia University Medical Center, New York, New York, United States of America; 7 Cell Circuits Program, Broad Institute, Cambridge, Massachusetts, United States of America; Nathan S Kline Institute, UNITED STATES

## Abstract

Alzheimer’s disease and related disorders (ADRD) may manifest cognitive and non-cognitive phenotypes including motor impairment, suggesting a shared underlying biology. We tested the hypothesis that five cortical proteins identified from a gene network that drives AD and cognitive phenotypes are also related to motor function in the same individuals. We examined 1208 brains of older adults with motor and cognitive assessments prior to death. Cortical proteins were quantified with SRM proteomics and we collected indices of AD and other related pathologies. A higher level of IGFBP5 was associated with poorer motor function proximate to death but AK4, HSPB2, ITPK1 and PLXNB1 were unrelated to motor function. The association of IGFBP5 with motor function was unrelated to the presence of indices of brain pathologies. In contrast, the addition of a term for cognition attenuated the association of IGFBP5 with motor function by about 90% and they were no longer related. These data lend support for the idea that unidentified cortical proteins like IGFBP5, which may not manifest a known pathologic footprint, may contribute to motor and cognitive function in older adults.

## 1. Introduction

There is increasing recognition that loss of cognitive and motor function in older adults are due in part to the accumulation of pathologies of Alzheimer’s disease and related disorders (ADRD) in older brains.[1] PET brain amyloid imaging and postmortem histopathology studies have shown that AD and related disorders such as Lewy bodies (LBs) and cerebrovascular disease disorders (CVD) are related to both cognitive and motor impairments highlighting that cognitive and motor impairments in older adults may share common underlying mechanisms.[1] However there is a paucity of data about cortical molecular mechanisms underlying impaired cognition and motor function in older adults.[2]

In a recent study we applied a system biology approach to RNAseq data from the dorsolateral prefrontal cortex (DLPFC) and identified a gene network (labeled module 109; m109)) consisting of 390 co-expressed genes that is a key driver of β-amyloid pathology and cognitive decline in older adults.[3] While this network is associated with cognitive decline its individual genes may have other clinical functions. Therefore in a further study, we employed selected reaction monitoring (SRM) targeted proteomics to validate twelve genes in this network predicted to control this cognitive gene network. We quantified protein abundance and found that protein levels of five of these twelve genes including IGFBP5 AK4, HSPB2, ITPK1 and PLXNB1 were associated with cognitive decline and/or AD pathology.[4]

Since cognitive and motor impairments may share a common biology, we tested the hypothesis that some of the five cortical proteins which drive impaired cognition might also be associated with poorer motor function in the same individuals. Then we examined if these associations were attenuated when terms for AD and other brain pathologies or cognition proximate to death were included in these models. To test these hypotheses we used clinical and postmortem data from older adults participating in two community-based cohort studies which both employ common data collection procedures and in which decedents undergo brain autopsy at the time of death.[5]

## 2. Materials and methods

### 2.1 Study participants

Participants were community dwelling older persons enrolled in one of two ongoing cohort studies of aging and dementia, the Religious Orders Study [ROS, N = 549 (45.5%] and Rush Memory and Aging Project (MAP, N = 659, 54.6%).[5] Both studies were approved by the Institutional Review Board of Rush University Medical Center. Participants entered the studies without known dementia and agreed to annual assessments as well as brain donation after death. A written informed consent and an anatomical gift act were obtained from each participant.

### 2.2 Assessment of motor function

We quantified ten motor tasks. (1&2) The Jamar hydraulic hand and pinch dynamometers (Lafayette Instruments, Lafayette) were employed to test bilateral grip and pinch strength. Dexterity of the arms was based on (3) the number of pegs placed in the Purdue Pegboard in thirty seconds. Two trials for each hand were averaged to provide a Purdue Pegboard score. (4) An electronic tapper (Western Psychological Services, Los Angeles, CA) was employed to determine how quickly participants were able to tap with their index finger for ten seconds. Two trials for each hand were averaged to yield a tapping score. We measured the (5, 6) time and (7, 8) number of steps taken to walk eight feet and turn 360^o^. (9) Participant’s stood on each leg for ten seconds to assess balance. (10) Then they were requested to stand on their toes for ten seconds.[6, 7]

A summary global motor score was constructed by scaling and averaging all ten tests. In prior studies, this measure was associated with diverse adverse health outcomes including survival, incident disabilities and incident cognitive impairment.[7, 8] To determine if different aspects of motor function were differentially associated with cortical proteins, we examined three motor abilities constructed from subsets of these ten measures including gait (4 tests), hand strength (2 tests) and manual dexterity (2 tests). These measures and their groupings are shown in **S1 Table**. We did not form a balance measure as sometimes it was not attempted.[6, 8]

### 2.3 Assessment of cognitive function

Each participant underwent annual medical history as well as structured cognitive and motor testing. Composite global cognition was derived from z scores of 17 cognitive tests employed for a harmonized measure of cognition for both cohorts. These measures are shown in **S1 Table**, using the baseline mean and SD and subsets of these same 17 tests were used to construct 5 different cognitive abilities as shown in **S1 Table**.[5, 9] Mini-Mental State Examination was assessed and used to describe the cohort but was not included in the global cognition score. The diagnosis of dementia and its causes was based on guidelines of the joint working group of the National Institute of Neurological and Communicative Disorders and Stroke and Alzheimer’s Disease and Related Disorders Association.[10]

### 2.4 Demographics

Age was computed from self-report date of birth and date of death. Sex and years of education were recorded at the study entry.

### 2.5 Assessment of AD and other brain pathology indices

Brain autopsy followed a standard protocol.[11] Neuropathologic evaluations, blinded to clinical data, assessed the burden of four common degenerative neuropathologies, including AD, Lewy bodies (LBs), nigral neuronal loss (NNL), TDP-43 and four cerebrovascular disease pathologies including cerebral infarcts, cerebral amyloid angiopathy (CAA), atherosclerosis and arteriolosclerosis.

Four degenerative brain pathologies included: *AD pathology*: Bielschowsky silver stain was used to visualize neuritic plaques, diffuse plaques, and neurofibrillary tangles in the frontal, temporal, parietal, and entorhinal cortex, and the hippocampus, as previously described.[12] We created standardized scores for each plaque and tangle count in each cortical area as previously described. These scaled scores for each region were then averaged across the five regions to develop summary scores for diffuse plaques, neuritic plaques, and neurofibrillary tangles for each subject. We then averaged the summary scores of the three AD markers to yield the global measure of AD pathology for each subject used in these analyses.[13] *Nigral neuronal loss* was assessed using a semi-quantitative scale (0–3) of a 6 micron section H&E stain in the substantia nigra near or at the exit of the 3rd nerve. *Lewy bodies* were treated as present or absent in analyses based on the exam of six regions (midfrontal cortex, superior or middle temporal cortex, inferior parietal cortex, anterior cingulate cortex, entorhinal cortex and substantia nigra) using a monoclonal phosphorylated antibody to a-synuclein (1:20,000; Wako Chemical USA Inc., Richmond, VA) with alkaline phosphatase as the chromogen. Monoclonal TDP-43 antibody was used to stage *TDP-43* from amygdala to limbic and neocortical regions.[14]

Four indices of cerebrovascular disease pathology included: The presence of *chronic macroscopic infarcts* were recorded during gross exam with subsequent histological confirmation.[15] Immunohistochemistry in four neocortical regions was employed to assess *cerebral amyloid angiopathy* (*CAA)*. A summary CAA measure was based on the average rating of β-amyloid depositions in the vessels of each of the four regions.[16] The severity of *atherosclerosis* was graded by gross examination of the vessels of the circle of Willis. A semi-quantitative scale was employed to grade the severity of *arteriolosclerosis* in the basal ganglia.[17]

### 2.6 Targeted SRM proteomics

The current study analyzes targeted proteomics data collected in a prior study from dorsolateral prefrontal cortex (DLPFC) using a standard protocol to prepare samples.[4, 18, 19] A 96 well plate format using Epmotion 5075 TMX (Eppendorf) or Liquidator96 (Rainin) was used to perform liquid handling. A denaturation buffer (8M urea, 50 mM Tris-HCl pH 7.5, 10 mM DTT, 1 mM EDTA) was used to homogenize about 20 mg of brain tissue from each individual. After denaturation and reduction, 400 μg protein aliquots were alkylated with 40 mM iodoacetamide and digested with trypsin (1:50 w/w trypsin to protein ratio). Solid phase extraction with Strata C18-E (55 μm, 70 Å) 25 mg/well 96-well plates (Phenomenex) on positive pressure manifold CEREX96 (SPEware) was employed to clean the digested samples. Tryptic peptide concentrations were readjusted to 1 μg/μL and 30 μL aliquots were mixed with 30 μL stable isotope-labeled synthetic peptides.

A nanoACQUITY UPLC was coupled to TSQ Vantage MS instrument and a sample injection of 2 μL was used for each of the measurements in the LC-SRM experiments. Buffer A used was 0.1% FA in water and buffer B was 0.1% in 90% ACN. Peptide separations were performed by an ACQUITY UPLC BEH 1.7 μm C18 column (75 μm i.d. × 25 cm) at a flow rate 350 nL/min using gradient of 0.5% of buffer B in 0–14.5 min, 0.5–15% B in 14.5–15.0 min, 15–40% B in 15–30 min and 45–90% B in 30–32 min.

**Fig 1:**
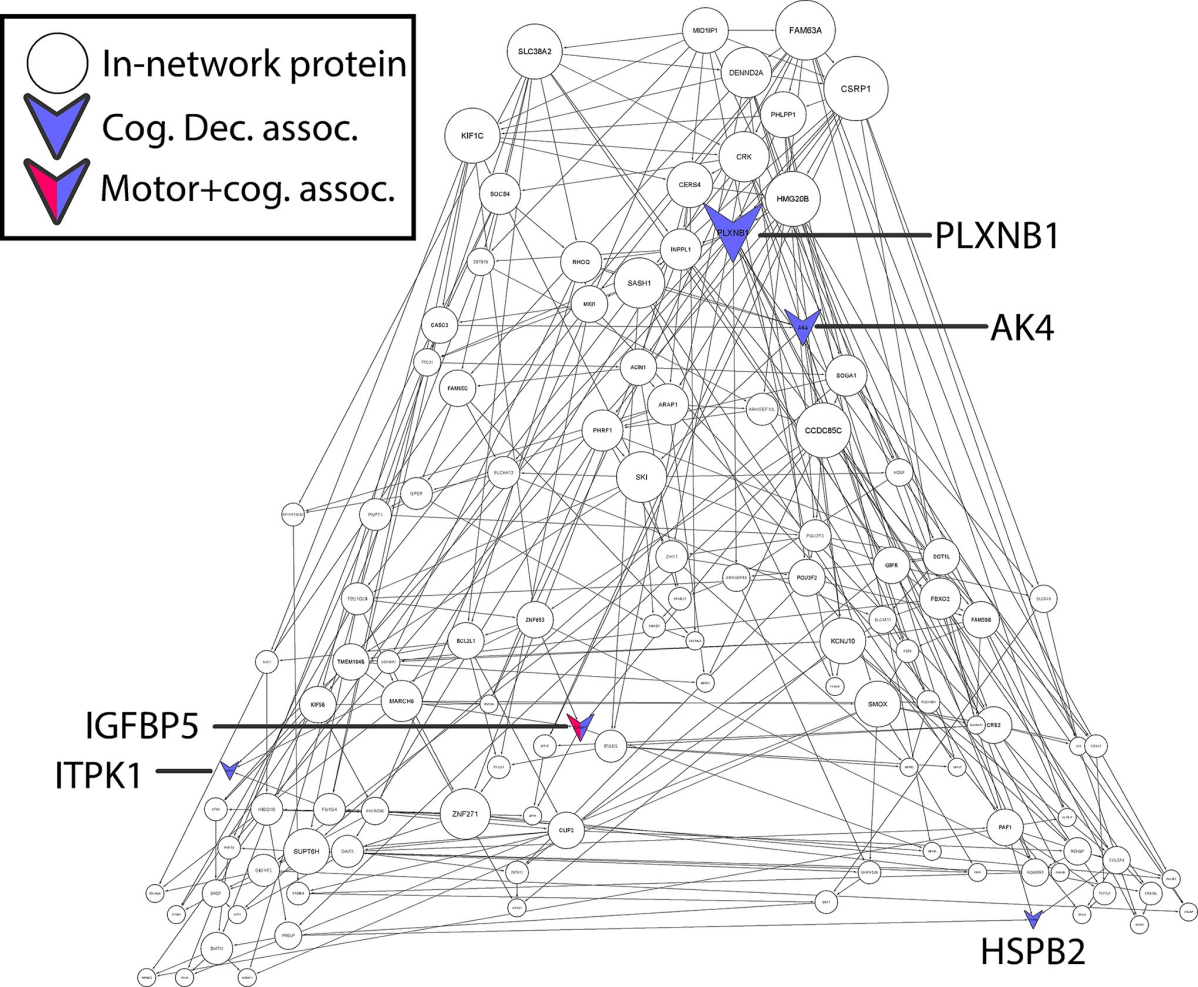
Cortical proteins in a cognitive frontal gene network are associated with both cognition and motor function in older adults. Shaded nodes are 12 proteins quantified with SRM to validate changes in levels of predicted influential genes in a network driving AD and cognitive decline. V-shaped nodes are cortical proteins associated with cognitive decline (N = 4), while IGFPB5 is related to both cognition and global motor score proximate to death (p < .05).

All the SRM data were manually inspected and analyzed with Skyline software [20]. For peptide quantification we excluded transitions interfering with co-eluting peptides. Skyline software automatically calculated the peak area ratios of endogenous light peptides and their heavy isotope-labeled internal standards (i.e., L/H peak area ratios). In case a protein was quantified with multiple peptides, we retained the peptide with the highest signal to noise ratio as the representative one. The peptide relative abundances (L/H ratios) were available from a prior study and these ratios were log base 2 transformed and centered at the median and these data were employed in the current analyses. **Fig 1**illustrates the location of the genes for the five proteins in the m109 coexpression network which were found to be associated with cognitive decline or AD pathology.[4]

### 2.7 Statistical analysis

We fit linear regression models with global motor score as the continuous outcome variable, and examined each of the five proteins previously shown to be associated with AD and cognitive decline. Unless otherwise noted, all the models controlled for age and sex. Then in a subsequent stage of analyses, we first examined which indices of 8 brain pathologies collected were independently associated with global motor score by including terms for all 8 indices together in a single model. Then we added a term for IGFPB5 together with indices of the four brain pathologies independently associated with global motor score to determine if these pathologies attenuated the association of cortical proteins with global motor score. Then, as the cortical proteins examined in this study have previously been shown to drive cognitive decline, we examined if including a term for cognition together with IGFPB5 attenuated the association of this protein with global motor score with and without indices of brain pathologies. Analyses were performed using SAS/STAT software (SAS Institute, Cary, NC).

## 3. Results

There were 1208 cases included in these analyses. Their clinical and postmortem characteristics are summarized in **Table 1**.

**Table 1:** Clinical and postmortem characteristics*.

MEASURE	N = 1208 Mean (SD); N (%)
**Clinical Measures**	
Age at Death (yrs)	89.4 (6.52)
Sex (% female)	822 (68.1)
Race Black	27 (2.2)
Non-hispanic White	1153 (95.4)
Education (yrs)	16.2 (3.59)
Clinical Dementia present	538 (45)
Mini-Mental Status Exam (0–30)	20.5 (9.36)
Global motor score (scaled average score)	0.67 (0.26)
Global cognitive score (z score)	-0.98 (1.21)
**Postmortem Measures**	
Interval from last visit to death (yrs)	0.9 (1.30)
Postmortem interval (hrs)	8.4 (6.01)
**Neurodegenerative**	
Nigral neuronal loss (mod-sev)	159 (13.2)
Lewy bodies present	322 (26.7)
NIA-Reagan diagnosis of AD	780 (64.6)
Global AD pathology	0.75 (0.63)
TDP-43	376 (32.6)
**Cerebrovascular Disease Pathologies**	
Macroscopic infarcts present	438 (36.3)
Atherosclerosis (mod-sev)	407 (33.8)
Arteriolosclerosis (mod-sev)	403 (33.6)
Cerebral amyloid angiopathy	430 (36.2)

*Cell entries are Mean (SD) or number (%)

### 3.1 Cortical proteins and global motor score

**Fig 2:**
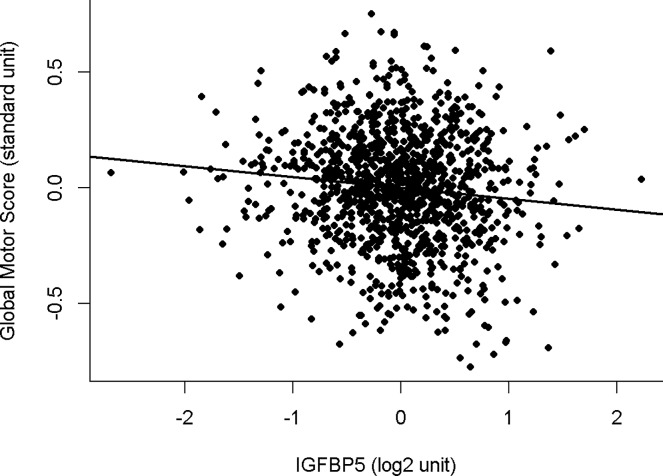
The association of IGFBP5 in the dorsal lateral prefrontal cortex with motor function proximate to death. This figure illustrates the association of residual global motor score (black line) with IGFBP5 controlling for age and sex.

A higher level of IGFBP5 was associated with poorer motor function proximate to death (**Table 2, Model 1). Fig 2**illustrates the association of IGFBP5 with level of motor function proximate to death. IGFBP5 was associated with each of the three motor abilities used to construct the global motor score: Strength: Estimate: -0.061 (S.E., 0.016, p = <0.001); Dexterity: Estimate: -0.089 (S.E., 0.014, p = <0.001) and Gait: Estimate: -0.055 (S.E., 0.017, p = <0.001).

**Table 2:** Association of IGFBP5, cognition, brain pathologies with global motor score proximate to death*.

Terms	Model 1 Estimate, (Standard Error, p-Value)	Model 2 Estimate, (Standard Error, p-Value)	Model 3 Estimate, (Standard Error, p-Value)	Model 4 Estimate, (Standard Error, p-Value)	Model 5 Estimate, (Standard Error, p-Value)	Model 6 Estimate, (Standard Error, p-Value)
**IGFBP5**	-0.047 (0.013,<0.001)		-0.038 (0.012,0.002)		-0.006 (0.012,0.634)	-0.005 (0.012,0.676)
**AD pathology**		-0.015 (0.020,0.460)				
**TDP-43**		0.010 (0.007,0.139)				
**Lewy bodies**		-0.025 (0.018,0.179)				
**Cerebral amyloid angiopathy**		-0.010 (0.008,0.234)				
**Macroinfarct**		-0.041 (0.015,0.008)	-0.039 (0.015,0.011)			-0.022 (0.015,0.143)
**Arteriolosclerosis**		-0.020 (0.008,0.021)	-0.019 (0.008,0.024)			-0.013 (0.008,0.099)
**Atherosclerosis**		-0.031 (0.009,0.001)	-0.030 (0.009,0.001)			-0.024 (0.009,0.006)
**Nigral Neuronal Loss**		-0.029 (0.010,0.004)	-0.032 (0.009,<0.001)			-0.015 (0.009,0.079)
**Cognition**				0.069 (0.006,<0.001)	0.068 (0.006,<0.001)	0.0609 (0.006,<0.001)

*Estimate, (Standard Error and p-Value) for a single regression model with terms for a single cortical protein and age and sex (not shown) with a motor outcome shown at the top of the column. The cortical protein **IGFBP5** was shown in a prior publication (reference 4) to be associated with cognitive decline.

In contrast, none of the other four proteins were associated with motor function [AK4: (Estimate, -0.040, S.E., 0.034, p = 0.238); HSPB2: (Estimate, -0.029, S.E., 0.016, p = 0.066); ITPK1: (Estimate, -0.005, S.E., 0.025, p = 0.850); PLXNB1: (Estimate, -0.032, S.E., 0.018, p<0.073)].

IGFBP5 was not associated with age of death or education and was similar in men and women (p’s>0.500). While almost half of the participants showed clinical dementia proximate to death, the association of IGFBP5 and motor function did not vary between individuals with and without dementia (IGFBP5 x Dementia, Estimate, -0.011, S.E., 0.025, p = 0.651).

### 3.2 Cortical proteins, AD and other brain pathologies and global motor score

For descriptive purposes we dichotomized the presence or absence of the postmortem indices measured as summarized in **Table 1**. The average participant showed evidence of 3 of the 8 brain pathologies collected (mean, 2.7, SD = 1.56) One or more pathologies were observed in nearly all cases (93.3%); 0: N = 81 (6.7%); 1: N = 202 (16.7%); 2: N = 263(21.8%); 3: N = 280 (23.2%); 4: N = 228 (18.9%); 5: N = 104 (8.6%); 6 or 7: N = 50, (4.1%).

First we examined which of the eight brain pathologies were independently associated with motor function proximate to death when included together in a single model. Four pathologies including: macroinfarcts, arteriolosclerosis, atherosclerosis and nigral neuronal loss were independently associated with global motor score, but AD, TDP-43, Lewy bodies pathologies and cerebral amyloid angiopathy were not related to global motor score (**Table 2, Model 2**).

Then we examined, if the association of IGFBP5 with global motor score was attenuated when terms for the 4 brain pathologies independently associated with motor function were included in the model. IGFBP5 remained independently associated with motor function when these indices of brain pathologies were included in the model (**Table 2, Model 3**). Similar findings were observed for each of the three motor abilities used to construct global motor score (results not shown).

**Fig 3:**
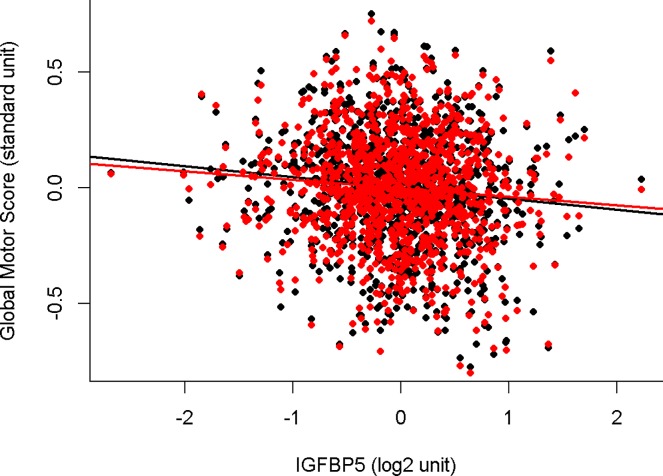
The association of IGFBP5 in the dorsal lateral prefrontal cortex with motor function proximate to death before and after controlling for brain pathologies. This figure illustrates the association of residual global motor score with IGFBP5 controlling for age and sex (black line average regression line and individual data points–black circles) and after controlling for demographics and brain pathologies (red line average regression line and individual data points–red circles). The slope of the black and red regression lines are nearly unchanged, suggesting that the association of IGFPB5 and motor function is unrelated to brain pathologies.

**Fig 3**illustrates the association of IGFBP5 with global motor score with and without terms for indices of brain pathologies associated with motor function. The regression line for the association of IGFBP5 with global motor score is nearly identical with (red line) and without (black line) terms for brain pathologies.

### 3.3 Cortical proteins, cognition and global motor score

**Fig 4:**
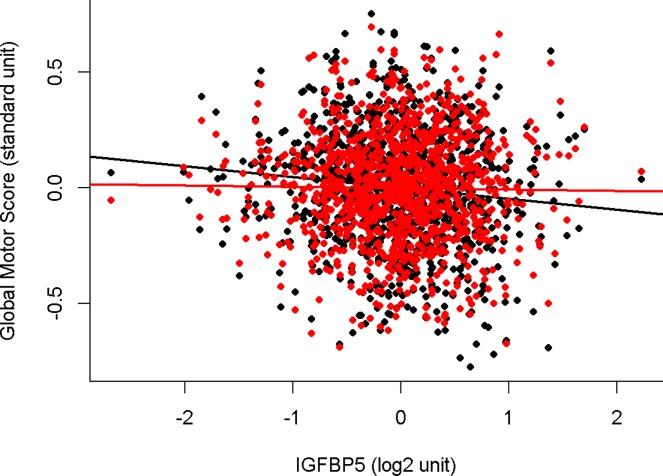
The association of IGFBP5 in the dorsal lateral prefrontal cortex with motor function proximate to death before and after controlling global cognition. This figure illustrates the association of residual global motor score with IGFBP5 controlling for age and sex (black line average regression line and individual data points–black circles) and after controlling for demographics and global cognition (red line average regression line and individual data points–red circles). After controlling for cognition, the slope of the red line is flat as compared to the black regression line, showing that IGFPB5 is no longer associated with motor function. This suggests that cognition may link IGFPB5 with motor function.

As we have shown in prior work, cognitive function was strongly associated with motor function proximate to death (**Table 2, Model 4**). Next we examined if the association of IGFBP5 and motor function was attenuated when a summary measure for cognitive function was included in the model. The association of IGFBP5 and motor function was attenuated by about 90% in the model which controlled for cognition and IGFBP5 was no longer associated with motor function (**Table 2, Model 5)**. These findings suggest that cognition may link (mediate) the association of IGFBP5 with motor function. **Fig 4**, illustrates that after controlling for cognition the slope of line for the association of IGFPB5 with motor function was nearly zero (red line) as compared to the black regression in the model without cognition because cognition severely attenuated the association of IGFBP5 with motor function and they were no longer related.

To insure that these findings were not driven by poor cognition in individuals with dementia proximate to death, we added an interaction term between IGFBP5 and global cognition score. The association of IGFBP5 and motor function did not vary with the global cognitive score (IGFBP5 x global cognitive score, Estimate, 0.003, S.E., 0.010, p = 0.772).

The attenuation of IGFBP5 with motor function in the presence of a term for global cognition was unchanged when postmortem indices are also included in the same model with cognition and IGFBP5 (**Table 2, Model 6**).

Global cognition is constructed from five different abilities so we examined if these different cognitive abilities each attenuated the association of IGFBP5 and motor function. The association of IGFBP5 and motor function was no longer significant when terms for the five cognitive abilities were included in the model and its estimate (**Table 3, Model 1**) was attenuated by 53%-87% (**Table 3, Models 2–7**). Working memory and perceptual speed showed the greatest attenuation of IGFPB5 association with motor function (**Table 3**).

**Table 3:** Association of IGFBP5, and cognitive abilities with global motor score proximate to death*.

Terms	Model 1 Estimate, (Standard Error, p-Value)	Model 2 Estimate, (Standard Error, p-Value)	Model 3 Estimate, (Standard Error, p-Value)	Model 4 Estimate, (Standard Error, p-Value)	Model 5 Estimate, (Standard Error, p-Value)	Model 6 Estimate, (Standard Error, p-Value)	Model 7 Estimate, (Standard Error, p-Value)
**IGFBP5**	-0.047 (0.013,<0.001)	-0.006 (0.012,0.634)	-0.022 (0.013,0.086)	-0.022 (0.012,0.067)	-0.010 (0.012,0.417)	-0.016 (0.012,0.188)	-0.014 (0.012,0.264)
**Global Cognition**		0.068 (0.006,<0.001)					
**Episodic Memory**			0.043 (0.005,<0.001)				
**Visual Spatial Abilities**				0.080 (0.007,<0.001)			
**Perceptual Speed**					0.074 (0.006,<0.001)		
**Semantic Memory**						0.057 (0.005,<0.001)	
**Working Memory**							0.065 (0.006,<0.001)
**% attenuation of IGFBP5**		87%	53%	53%	79%	66%	70%

*Each cell shows the Estimate, (Standard Error and p-Value) for a single regression model with terms shown in the left column as well as age and sex (not shown). The outcome for all the models is global motor score proximate to death.

In further analyses, we repeated the previous models adding interaction terms, global cognition did not modify the association of IGFBP5 with motor function (IGFBP5 x Global cognition: (Estimate: 0.003,S.E., 0.010, p = 0.772). In similar models, the association of IGFBP5 and motor function proximate to death did not vary with any of the five cognitive abilities (results not shown).

## 4. Discussion

There is a paucity of data about molecular pathways which underlie late-life motor impairment. In a prior study, we identified five cortical proteins from a frontal gene network which underlie cognitive decline and AD in older adults.[3, 4] In the current study, we extend these findings to show that IGFBP5, one of these five cortical proteins is also related to motor function in these same adults. The association of IGFBP5 with motor function was unrelated to AD and other brain pathologies. In contrast, the association of IGFBP5 with motor function was almost completely attenuated when we controlled cognitive function. This suggests that cognition may link cortical proteins, without a pathologic footprint, which may drive late-life motor impairment. These novel data offer specific gene and protein targets that have potential to inform on the complex shared biology underlying late-life cognitive and motor impairment and facilitate the development of strategies to maintain or prevent their decline in older adults.

Prior brain imaging and postmortem studies suggest that like cognition, impaired mobility is related to AD and other related brain pathologies (ADRD), and that the level and rates of declining cognitive and motor function are correlated. These data suggest that motor and cognitive function may share a common pathologic basis, but the molecular pathways underlying their shared decline remain to be identified. In prior work we employed a novel systems biology approach to leverage whole transcriptome data from the dorsolateral prefrontal cortex (DLPFC) and identified twelve genes which control a cognitive gene network. ^20^ Further work using SRM targeted proteomics validated that cortical proteins levels of five of these genes drive AD pathology and cognitive decline in older adults.[3]

The current work extends this prior SRM study by showing that cognition and motor function in older adults may share not only common brain pathologies, but may also share common cortical proteins and molecular pathways driving their decline. Since the full set of proteins studied with SRM was derived from a larger list of genes chosen as likely drivers of a cognitive gene network, it may account for why only one protein was associated with motor function.[3] Further work applying a similar approach to other gene networks in the prefrontal cortex and other brain-related motor regions is likely to identify motor-related gene networks and a larger number of motor-specific genes and molecular pathways which may also be shared with cognition.

In this study while IGFPB5 was related to motor function it was linked to motor function via cognitive function. Conventional measures of motor performance focus on the characterization of discernable movement and action. Volitional action or movement in human is a complex behavior which requires the orchestration of interconnected cognitive and motor neural systems which control gait initiation, planning and execution and which are active prior to any discernable movement.[21–27] Moreover, even after movement begins additional cognitive-motor neural systems are crucial to adapt movements to meet motivational and environmental demands. These features of movement underscore the prominent role of cognition in all movement.

The current findings suggest a potential casual molecular pathway underlying inter-related cognitive and motor function in movement. Our approach and novel finding has potential to allow investigators to move beyond descriptions and simple associations between cognitive and motor function in older adults. Further studies could target this pathway to explicate the mechanisms underlying the integrated biology of cognition and motor function driving movement. Moreover, therapies targeting molecular pathways common to both cognitive and motor function have the potential to ameliorate multiple declining faculties with a single intervention. Since poor motor function may precede and predict incident cognitive impairment in older adults and impaired cognition may occur in older adults with normal motor function, it is likely that further investigations will identify molecular pathways specific for cognition or motor function in addition to pathways common to both cognition and motor function.

Due to the unique study design of the cohorts examined in this study, postmortem indices of ADRD pathologies were also available and examined in our analyses. Recent work has suggested that like AD dementia, late-life motor impairment is most commonly associated with indices of mixed brain ADRD pathologies. Thus, while indices of mixed ADRD brain pathologies have consistently been associated with motor function in older adults, the specific pathologies associated with motor function have varied in these studies. [6, 28–34] While AD pathology was not related to motor function in the current study, indices of four other ADRD pathologies were associated with motor function proximate to death in the current study. Although AD and other brain pathologies are related to both motor and cognitive function in old adults, the causal pathway linking IGFBP5 with motor function in this study was unaffected when we added terms to control for indices of ADRD pathologies. These findings add to our prior work which has shown that other clinical and biologic risk factors related to cognitive or motor phenotypes are unrelated to indices of ADRD brain pathologies.[35–37] These findings underscore the importance of complementing effort to characterize histopathology underlying decline with approaches which can identify molecular pathways that may drive late-life motor and cognitive impairment without a known pathologic footprint.[28, 38]

This study has several limitations. Transcriptomic and proteomic data came from a single brain region supporting a cognitive gene network. However, cortical proteins and histopathology collected from cognitive brain regions may only account for a minority of late-life motor impairment.[28, 38] In contrast to cognition, the neural control systems underlying movement extend beyond the brain to spinal cord and via the peripheral nervous system to muscle, the final effector of all movement. Thus, to more fully explicate the molecular pathways underlying impaired motor function will require interrogation of brain as well as key motor tissues outside the brain. The cohorts studied are voluntary cohorts, and participants were older and highly educated. As such, generalizability of these findings rely on replication in samples from other longitudinal cohorts. The study also has several strengths. Findings are based on a large sample of well-characterized autopsied individuals. SRM proteomics technique, in combination with structured motor and cognitive evaluations prior to death and comprehensive pathologic assessments, provide unique high-quality multi-level data from the same individuals to perform robust statistical modeling.

In summary, prior histopathology studies suggest that late-life cognitive and motor function are related to common age-related pathologies. The current study extends these histopathology findings by showing that both cognition and motor function are also related to a cortical protein, IGFBP5 which may not manifest a known pathologic footprint. Further work extending the novel system biology methods employed to identify cognitive gene networks to additional key motor tissues may facilitate the identification of a host of molecular pathways and provide novel targets for further studies to maintain both cognition and motor function in older adults.

## Supporting information

S1 Table Cognitive and motor measures used to construct composite scores. (DOCX)

## References

[1] Albers MW, Gilmore GC, Kaye J, Murphy C, Wingfield A, Bennett DA, et al At the interface of sensory and motor dysfunctions and Alzheimer's disease. Alzheimers Dement. 2015;11(1):70–98. Epub 2014/07/16. 10.1016/j.jalz.2014.04.514 25022540 PMC4287457

[2] Rosso AL, Studenski SA, Chen WG, Aizenstein HJ, Alexander NB, Bennett DA, et al Aging, the Central Nervous System, and Mobility. The Journals of Gerontology Series A: Biological Sciences and Medical Sciences. 2013;68:1379–86. 10.1093/gerona/glt089 23843270 PMC3805295

[3] Mostafavi S, Gaiteri C, Sullivan SE, White CC, Tasaki S, Xu J, et al A molecular network of the aging human brain provides insights into the pathology and cognitive decline of Alzheimer's disease. Nat Neurosci. 2018;21(6):811–9. Epub 2018/05/29. 10.1038/s41593-018-0154-9 . 29802388 PMC6599633

[4] Yu L, Petyuk VA, Gaiteri C, Mostafavi S, Young-Pearse T, Shah RC, et al Targeted brain proteomics uncover multiple pathways to Alzheimer's dementia. Ann Neurol. 2018;84(1):78–88. Epub 2018/06/17. 10.1002/ana.25266 29908079 PMC6119500

[5] Bennett DA, Buchman AS, Boyle PA, Barnes LL, Wilson RS, Schneider JA. Religious Orders Study and Rush Memory and Aging Project. J Alzheimers Dis. 2018;64:S161–S89. Epub 2018/06/06. 10.3233/JAD-179939 . 29865057 PMC6380522

[6] Buchman AS, Yu L, Boyle PA, Levine SR, Nag S, Schneider JA, et al Microvascular Brain Pathology and Late-Life Motor Impairment. Neurology. 2013;80:712–8. 10.1212/WNL.0b013e3182825116 23365057 PMC3589297

[7] Buchman AS, Leurgans SE, Boyle PA, Schneider JA, Arnold SE, Bennett DA. Combinations of Motor Measures More Strongly Predict Adverse Health Outcomes in Old Age: The Rush Memory and Aging Project, a Community-Based Cohort Study. BMC Medicine. 2011;9:42 10.1186/1741-7015-9-42 21507235 PMC3100235

[8] Wilson RS, Segawa E, Buchman AS, Boyle PA, Hizel LP, Bennett DA. Terminal Decline in Motor Function. Psych & Aging. 2012;4:988–1007. 10.1037/a0028182 PMC3480971 22612603

[9] Wilson RS, Boyle PA, Yu L, Segawa E, Sytsma J, Bennett DA. Conscientiousness, dementia related pathology, and trajectories of cognitive aging. Psychol Aging. 2015;30(1):74–82. Epub 2015/02/11. 10.1037/pag0000013 25664558 PMC4361241

[10] Bennett DA, Schneider JA, Buchman AS, Barnes LL, Boyle PA, Wilson RS. Overview and Findings From the Rush Memory and Aging Project. Curr Alzheimer Res. 2012;9:646–63. Epub 2012/04/05. . 22471867 10.2174/156720512801322663 PMC3439198

[11] Schneider JA, Arvanitakis Z, Leurgans SE, Bennett DA. The neuropathology of probable Alzheimer disease and mild cognitive impairment. Annals of neurology. 2009;66(2):200–8. 10.1002/ana.21706 19743450 PMC2812870

[12] Bennett DA, Schneider JA, Arvanitakis Z, Kelly JF, Aggarwal NT, Shah RC, et al Neuropathology of older persons without cognitive impairment from two community-based studies. Neurology. 2006;66(12):1837–44. Epub 2006/06/28. 10.1212/01.wnl.0000219668.47116.e6 . 16801647

[13] Bennett DA, Schneider JA, Wilson RS, Bienias JL, Arnold SE. Neurofibrillary tangles mediate the association of amyloid load with clinical Alzheimer disease and level of cognitive function. Arch Neurol. 2004;61(3):378–84. Epub 2004/03/17. 10.1001/archneur.61.3.378 . 15023815

[14] Yu L, De Jager PL, Yang J, Trojanowski JQ, Bennett DA, Schneider JA. The TMEM106B locus and TDP-43 pathology in older persons without FTLD. Neurology. 2015;84(9):927–34. Epub 2015/02/06. 10.1212/WNL.0000000000001313 25653292 PMC4351662

[15] Schneider JA, Bienias JL, Wilson RS, Berry-Kravis E, Evans DA, Bennett DA. The apolipoprotein E epsilon4 allele increases the odds of chronic cerebral infarction [corrected] detected at autopsy in older persons. Stroke. 2005;36(5):954–9. Epub 2005/03/19. 10.1161/01.STR.0000160747.27470.2a . 15774818

[16] Yu L, Boyle PA, Nag S, Leurgans S, Buchman AS, Wilson RS, et al APOE and cerebral amyloid angiopathy in community-dwelling older persons. Neurobiol Aging. 2015;36(11):2946–53. Epub 2015/09/06. 10.1016/j.neurobiolaging.2015.08.008 26341746 PMC4609607

[17] Arvanitakis Z, Capuano AW, Leurgans SE, Bennett DA, Schneider JA. Relation of cerebral vessel disease to Alzheimer's disease dementia and cognitive function in elderly people: a cross-sectional study. Lancet Neurol. 2016;15(9):934–43. Epub 2016/06/18. 10.1016/S1474-4422(16)30029-1 27312738 PMC4969105

[18] Petyuk VA, Qian WJ, Smith RD, Smith DJ. Mapping protein abundance patterns in the brain using voxelation combined with liquid chromatography and mass spectrometry. Methods (San Diego, Calif). 2010;50(2):77–84. Epub 2009/08/06. 10.1016/j.ymeth.2009.07.009 19654045 PMC2818068

[19] Andreev VP, Petyuk VA, Brewer HM, Karpievitch YV, Xie F, Clarke J, et al Label-free quantitative LC-MS proteomics of Alzheimer's disease and normally aged human brains. Journal of proteome research. 2012;11(6):3053–67. Epub 2012/05/09. 10.1021/pr3001546 22559202 PMC3445701

[20] MacLean B, Tomazela DM, Shulman N, Chambers M, Finney GL, Frewen B, et al Skyline: an open source document editor for creating and analyzing targeted proteomics experiments. Bioinformatics. 2010;26(7):966–8. Epub 2010/02/12. 10.1093/bioinformatics/btq054 20147306 PMC2844992

[21] Richard A, Van Hamme A, Drevelle X, Golmard JL, Meunier S, Welter ML. Contribution of the supplementary motor area and the cerebellum to the anticipatory postural adjustments and execution phases of human gait initiation. Neuroscience. 2017;358:181–9. Epub 2017/07/05. 10.1016/j.neuroscience.2017.06.047 . 28673716

[22] Nachev P, Kennard C, Husain M. Functional role of the supplementary and pre-supplementary motor areas. Nat Rev Neurosci. 2008;9(11):856–69. 10.1038/nrn2478 18843271

[23] Jacobs JV, Lou JS, Kraakevik JA, Horak FB. The supplementary motor area contributes to the timing of the anticipatory postural adjustment during step initiation in participants with and without Parkinson's disease. Neuroscience. 2009;164(2):877–85. Epub 2009/08/12. 10.1016/j.neuroscience.2009.08.002 19665521 PMC2762010

[24] Hikosaka O, Isoda M. Switching from automatic to controlled behavior: cortico-basal ganglia mechanisms. Trends in Cognitive Sciences. 2010;14(4):154–61. 10.1016/j.tics.2010.01.006 20181509 PMC2847883

[25] Hikosaka O, Nakamura K, Sakai K, Nakahara H. Central mechanisms of motor skill learning. Curr Opin Neurobiol. 2002;12(2):217–22. . 12015240 10.1016/s0959-4388(02)00307-0

[26] Benarroch EE. Pedunculopontine nucleus: functional organization and clinical implications. Neurology. 2013;80(12):1148–55. Epub 2013/03/20. 10.1212/WNL.0b013e3182886a76 . 23509047

[27] Rothwell JC. Overview of neurophysiology of movement control. Clin Neurol Neurosurg. 2012;114(5):432–5. Epub 2012/01/28. 10.1016/j.clineuro.2011.12.053 . 22280985

[28] Buchman AS, Dawe RJ, Yu L, Lim A, Wilson RS, Schneider JA, et al Brain pathology is related to total daily physical activity in older adults. Neurology. 2018;90(21):e1911–e9. Epub 2018/04/27. 10.1212/WNL.0000000000005552 29695600 PMC5962918

[29] Buchman AS, Wilson RS, Shulman JM, Leurgans SE, Schneider JA, Bennett DA. Parkinsonism in Older Adults and Its Association With Adverse Health Outcomes and Neuropathology. J Gerontol A Biol Sci Med Sci. 2016;71(4):549–56. Epub 2015/09/13. 10.1093/gerona/glv153 26362440 PMC5014188

[30] Buchman AS, Yu L, Wison RS, Dawe R, VanderHorst V, Schneider JA, et al Post-mortem brain pathology is related to declining respiratory function in community-dwelling older adults. Frontiers in Aging Neuroscience. 2015;7 10.3389/fnagi.2015.00197 26539108 PMC4612667

[31] Buchman AS, Yu L, Wilson RS, Schneider JA, Bennett DA. Association of brain pathology with the progression of frailty in older adults. Neurology. 2013;80(22):2055–61. Epub 2013/05/03. 10.1212/WNL.0b013e318294b462 23635961 PMC3716398

[32] Buchman AS, Shulman JM, Nag S, Leurgans SE, Arnold SE, Morris MC, et al Nigral pathology and parkinsonian signs in elders without Parkinson disease. Ann Neurol. 2012;71(2):258–66. Epub 2012/03/01. 10.1002/ana.22588 22367997 PMC3367476

[33] Buchman AS, Yu L, Wilson RS, Boyle PA, Schneider JA, Bennett DA. Brain pathology contributes to simultaneous change in physical frailty and cognition in old age. J Gerontol A Biol Sci Med Sci. 2014;69(12):1536–44. Epub 2014/08/20. 10.1093/gerona/glu117 25136002 PMC4296120

[34] Buchman AS, Yu L, Wilson RS, Leurgans SE, Nag S, Shulman JM, et al Progressive parkinsonism in older adults is related to the burden of mixed brain pathologies. Neurology. 2019;92(16):e1821–e30. Epub 2019/03/22. 10.1212/WNL.0000000000007315 . 30894446 PMC6550497

[35] Wilson RS, Boyle PA, Yu L, Barnes LL, Schneider JA, Bennett DA. Life-span cognitive activity, neuropathologic burden, and cognitive aging. Neurology. 2013;81(4):314–21. Epub 2013/07/05. 10.1212/WNL.0b013e31829c5e8a 23825173 PMC3772831

[36] Wilson RS, Krueger KR, Arnold SE, Schneider JA, Kelly JF, Barnes LL, et al Loneliness and risk of Alzheimer disease. Arch Gen Psychiatry. 2007;64(2):234–40. Epub 2007/02/07. 10.1001/archpsyc.64.2.234 . 17283291

[37] Canli T, Yu L, Yu X, Zhao H, Fleischman D, Wilson RS, et al Loneliness five years ante-mortem is associated with disease-related differential gene expression in postmortem dorsolateral prefrontal cortex. Translational psychiatry. 2018;In Press. 10.1038/s41398-017-0086-2 PMC5802527 29317593

[38] Buchman AS, Leurgans SE, Nag S, VanderHorst V, Kapasi A, Schneider JA, et al Spinal Arteriolosclerosis Is Common in Older Adults and Associated With Parkinsonism. Stroke. 2017;48(10):2792–8. Epub 2017/09/22. 10.1161/STROKEAHA.117.017643 28931619 PMC5659359

