# Supplementary material for: Cognition may link cortical IGFBP5 levels with motor function in older adults
Source: PLoS One. 2019 Aug 12;14(8):e0220968. doi: 10.1371/journal.pone.0220968 (PMC6690580; doi:10.1371/journal.pone.0220968)
Supplement: S1 Table — (DOCX) [file pone.0220968.s001.docx]

**S1 TABLE. COGNITIVE AND MOTOR MEASURES USED TO CONSTRUCT COMPOSITE SCORES**

| **GLOBAL COGNITION SCORE** | | |
| --- | --- | --- |
| **TEST** | **COGNITIVE TEST SCORES** | **COGNITIVE ABILITIES** |
| **1** | Word List | **Episodic Memory** |
| **2** | Word List Recall |  |
| **3** | Word List Recognition |  |
| **4** | East Boston immediate recall |  |
| **5** | East Boston delayed recall |  |
| **6** | Logical memory I (immediate) |  |
| **7** | Logical memory I (immediate) |  |
| **8** | Boston naming (15 items) | **Semantic Memory** |
| **9** | Category fluency |  |
| **10** | Reading test - (10 items) |  |
| **11** | Digits forward | **Working Memory** |
| **12** | Digits backward |  |
| **13** | Digit ordering |  |
| **14** | Line orientation | **Visuospatial abilities** |
| **15** | Progressive matrices (16 items) |  |
| **16** | Symbol digits modality-oral | **Perceptual Speed** |
| **17** | Number comparison |  |

| **GLOBAL MOTOR SCORES** | | |
| --- | --- | --- |
| **TEST** | **MOTOR PERFORMANCE MEASURES** | **MOTOR ABILITIES** |
| **1** | Purdue Pegboard Test (no. of pegs) | **Motor Dexterity** |
| **2** | Finger-tapping test (taps/10 seconds) |  |
| **3** | Time to cover a distance of 8 feet (seconds) | **Motor Gait** |
| **4** | Number of steps required to cover 8 feet (steps) |  |
| **5** | 360 degree turn time (seconds) |  |
| **6** | Number of steps to complete a 360 degree turn (steps) |  |
| **7** | Leg stand (seconds) |  |
| **8** | Toe stand (seconds) |  |
| **9** | Grip strength (kilograms) | **Motor Strength** |
| **10** | Pinch strength (kilograms) |  |

Raw scores from a battery of cognitive tests were converted to Z scores and averaged to yield a global cognitive function summary. Mean and standard deviation at baseline were used to compute the z-scores. Z-score has mean 0 and standard deviation of 1. Each z-score corresponds to a point in a normal distribution. Z-score describes how much a point deviates from a mean or specific point. A negative z-score simply means that someone has an overall score that is lower than the average of the entire cohort at baseline.
